# Quantifying carotid plaque component regional strain using ultrasound elastography and its association with blood pressure in patients: a pilot study

**DOI:** 10.3389/fbioe.2026.1817145

**Published:** 2026-06-09

**Authors:** Jian Cai, Feihong Yu, Shumei Miao, Yang Xu, Huangwen Yang, Heming Chen, Rui Lv, Qingyu Wang

**Affiliations:** 1 School of Biomedical Engineering and Informatics, Nanjing Medical University, Nanjing, China; 2 The First Affiliated Hospital of Nanjing Medical University, Nanjing Medical University, Nanjing, China; 3 Institute of Medical Informatics and Management, Nanjing Medical University, Nanjing, China; 4 School of Health Science and Engineering, University of Shanghai for Science and Technology, Shanghai, China; 5 Department of Cardiovascular Surgery, Shandong Second Provincial General Hospital, Shandong University, Jinan, China

**Keywords:** carotid artery, optical flow method, plaque component, strain calculation, ultrasound elastography

## Abstract

Cardiovascular and cerebrovascular diseases are leading causes of mortality worldwide, with carotid plaque rupture being a critical pathogenic mechanism. While ultrasound elastography can assess the mechanical properties of plaque, the dynamic strain of specific plaque component regions and its correlation with blood pressure are not well characterized. This pilot study retrospectively analyzed carotid ultrasound elastography images from 11 patients. Image preprocessing involved wavelet Bayesian denoising and enhanced correlation coefficient registration to minimize motion artifacts. Dense optical flow was computed using the Farneback algorithm, and regional strain was derived through spatial gradient extraction and least-squares fitting. Our analysis revealed substantial heterogeneity in strain among plaque component regions. The mean inter-frame strain ranged from 0.0007 to 0.0045, the maximum inter-frame strain from 0.0025 to 0.0454, and the maximum cumulative strain over the cardiac cycle from 0.0078 to 0.1948. Diastolic blood pressure showed positive correlations with the mean inter-frame strain, the maximum cumulative strain and the maximum inter-frame strain of plaque component regions (r = 0.5121, 0.4606, 0.3977), the pulse pressure demonstrated moderate negative correlations with all three strain indices (r = −0.5043, −0.4757, −0.4650), showing statistical significance even with a small sample size. These exploratory findings suggest that blood pressure may influence the mechanical environment of plaque component regions. This pilot study validates the feasibility of dynamic strain quantification for plaque component regions using ultrasound elastography. The identified association with blood pressure provides new directions for investigating mechanical factors in plaque progression and developing non-invasive risk prediction models.

## 1 Introduction

Cardiovascular and cerebrovascular diseases are major global health threats, characterized by high prevalence, disability, and mortality rates (Neal et al., 2021). Carotid atherosclerosis is a key etiological factor in cerebrovascular diseases, including stroke and transient ischemic attacks. The pathogenesis begins with intimal lipid accumulation, progressing through fibrous tissue hyperplasia and calcification. Advanced plaques lead to arterial stenosis, impairing blood flow, while the development of vulnerable plaques-characterized by thin fibrous caps, large lipid cores, and inflammatory infiltration-significantly increases the risk of rupture (North American Symptomatic Carotid Endarterectomy Trial Collaborators et al., 1991). Subsequent thrombosis can cause vessel occlusion or embolization, culminating in acute clinical events. Thus, accurate assessment of plaque vulnerability and rupture risk is essential for disease prevention and treatment (Naghavi et al., 2003; Hossain et al., 2024).

Conventional imaging techniques such as ultrasound, computed tomography (CT), and magnetic resonance imaging (MRI) have shown some utility in plaque detection. However, the high cost of MRI and the ionizing radiation of CT limit their suitability for routine screening of vulnerable plaques. It is important to note that the mechanical environment of a plaque is not isolated but is continuously influenced by hemodynamic factors, such as blood pressure (BP). Theoretically, as the primary load on the vessel wall, variations in BP may directly modulate the stress-strain distribution within the plaque, thereby affecting its stability and progression.

Ultrasound elastography (UE) has emerged as a non-invasive solution for estimating tissue elastic properties and has been widely applied in vascular physiology and pathophysiology (Hansen et al., 2016; Chen et al., 2024). Numerous methodologies for strain calculation from dynamic imaging provide a foundation for carotid plaque analysis. Zhu et al., proposed a one-dimensional radial strain computation approach using optical flow and cross-correlation method combined with a Savitzky-Golay differential filter to compute displacement and vessel wall strain in intravascular ultrasound elastography (Zhu et al., 2009). Aggarwal et al. utilized 4D echocardiographic images and inverse finite element modeling to investigate the viscoelastic properties of cardiovascular tissues, the influence of perivascular structures on aortic wall mechanics, and global longitudinal strain measurements for assessing left ventricular function (Aggarwal et al., 2023). Zhang et al. reviewed three commonly used *in vivo* strain quantification techniques-implantable strain sensors, virtual fiber elongation, and ultrasound-noting that ultrasound elastography, which employs dynamic planar imaging to measure soft tissue deformation, can be applied in myocardial strain analysis (Zhang et al., 2021). Anderson et al. explored computational approaches for assessing fracture repair, developing patient-specific mechanobiological models to analyze strain fields within the fracture gap. This enables more accurate correlation between strain and healing outcomes, prediction of the healing process, and support for personalized treatment planning (Anderson et al., 2014). With ongoing advancements and expanding applications, ultrasound elastography holds broad prospects in medical imaging and demonstrates significant clinical potential in the evaluation and prevention of carotid plaque. However, current research on the quantitative analysis of strain at the component level of carotid plaques remains limited. Studies that correlate component-specific strain with key BP parameters are even scarcer, which hampers a comprehensive understanding of the plaque mechanical micro-environment and poses challenges for clinical translation.

This study aims to address this gap by dynamically quantifying strain within carotid plaque component regions using ultrasound elastography combined with an optical flow method. We enhanced image quality through wavelet transform denoising and motion compensation via an enhanced correlation coefficient registration algorithm. The Farneback dense optical flow algorithm was employed to track inter-frame displacement fields, and a region of interest (ROI) feature point update mechanism was incorporated. Spatial gradients were derived and least squares fitting was applied to obtain overall regional strain (Farneback et al., 2003). Furthermore, the correlation between carotid plaque strain and BP was investigated to identify potential early warning indicators for cerebrovascular events. Plaque component regional strain results are visualized using heatmaps and time–strain curves, facilitating clinical interpretation and display. This approach assists in assessing plaque rupture risk, improves the identification of vulnerable plaques, and offers a reference for individualized risk assessment. The present work helps fill a gap in the quantitative strain analysis of plaque component regions. The data is derived from patients diagnosed with type 2 diabetes. The correlation results between carotid plaque component regional strain and blood pressure are helpful for future determination of new warning indicators for cerebrovascular events and for guiding the medication intensity of patients with discovered plaques, which has significant clinical applicability.

## 2 Materials and methods

### 2.1 Study population and clinical data collection

This study was approved by the Ethics Committee of Nanjing Medical University, and informed consents were obtained from the patients. All patient data were anonymized. We retrospectively enrolled 11 patients with each patient presenting 1 to 3 carotid plaques and a confirmed diagnosis of type 2 diabetes who underwent carotid ultrasound at the First Affiliated Hospital of Nanjing Medical University between January 2023 and January 2024. The ultrasound images were acquired during routine carotid artery ultrasound examinations. Demographic and clinical history data were retrieved from the electronic medical record system.

**FIGURE 1:**
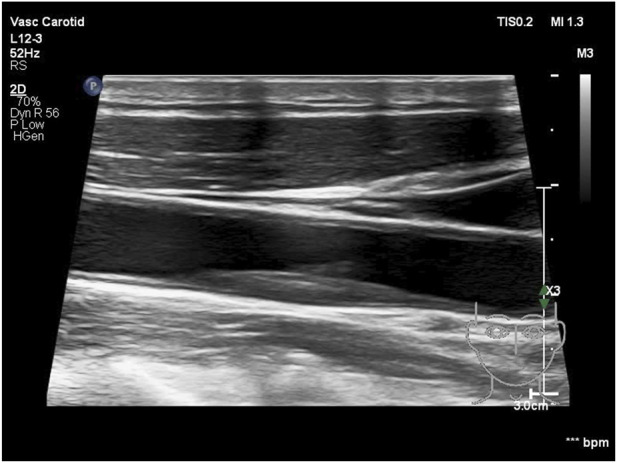
Axial dynamic ultrasound image of the carotid artery bifurcation.

Inclusion criteria were: (1): age ≥18 years; (2); diagnosis of type 2 diabetes; (3); clear ultrasound image quality allowing definitive identification of plaque component regions, shown in Figure 1; (4); complete cuff blood pressure measurement records on the day of examination; and (5) availability of complete clinical biomarker data. Exclusion criteria included: (1): images with significant artifacts that severely hampered plaque analysis; (2); factors leading to instability in image sequence periodicity, such as severe arrhythmia; and (3) history of ipsilateral carotid surgery or interventional therapy.

All carotid ultrasound examinations were performed by a single operator with over 5 years of vascular ultrasound experience, who was blinded to the specific hypotheses of this study. During the examination, patients were placed in a supine position with the head slightly extended and turned to the opposite side. The probe was gently placed on the neck to avoid external compression of the vessel. Long-axis dynamic ultrasound images of the carotid bifurcation were acquired, and high-quality cine loops encompassing at least three complete cardiac cycles were stored for offline analysis.

### 2.2 Image preprocessing

The acquired carotid ultrasound cine loops underwent denoising using a wavelet transform-based BayesShrink soft-thresholding method (Chang et al., 2000). In ultrasound elastography, motion artifacts arising from probe manipulation or patient physiological movements (e.g., respiration) can compromise the accuracy of subsequent strain calculations based on optical flow. To mitigate this, image registration of consecutive frames was performed to eliminate these artifacts, ensuring pixel-to-pixel correspondence of the same tissue points between adjacent frames and providing a reliable foundation for subsequent strain computation and quantitative analysis. An iterative optimization algorithm based on the Enhanced Correlation Coefficient (ECC) metric was employed for this purpose, utilizing the findTransformECC interface in OpenCV.

### 2.3 Region of interest tracking via displacement field

The Farneback algorithm was used to compute the dense displacement field between adjacent frames of the ultrasound image sequence. The calcOpticalFlowFarneback function in OpenCV was called, which implements Gunnar Farneback’s polynomial expansion algorithm within a multi-level pyramid framework to calculate the dense optical flow field between the previous and current frames. The output is an array of the same size as the input image, where each element contains the displacement components in the vertical (u) and horizontal (v) directions (Shi et al., 2002).

The definition of region of interest (ROI) is based on the established ultrasonic echo standards for the plaque component regions that are rich in lipids, fibrous, and calcified areas. ROI was manually selected by defining a polygon via mouse clicks on the first frame, which was then converted to floating-point coordinates. This manual process ensured precise alignment of the ROI with the carotid plaque component boundaries.

Within the ROI mask, Shi-Tomasi corner points were detected as initial feature points for tracking. The Lucas-Kanade sparse optical flow algorithm was applied to track these ROI polygon vertices dynamically. The new positions of the feature points were computed, and invalid tracks were filtered out based on status flags. Only validly tracked points were used to update the coordinates, thereby preventing ROI distortion due to erroneous matches. The median displacement of the valid feature points was applied as a global translation to all polygon vertices, enabling dynamic ROI tracking (Lucas et al., 1981).

To prevent feature loss from occlusion and to mitigate cumulative drift inherent in long-term tracking, a re-detection mechanism was implemented where feature points within the current ROI were refreshed every 30 frames. This approach, combined with the mask constraint that confines detection to the ROI, ensured a sufficient number of features for stable translation estimation throughout long image sequences.

### 2.4 Plaque component regional strain calculation

Based on the dense displacement field estimated by the Farneback algorithm, the spatial gradients of the displacement field were computed using the Sobel gradient operator, as defined by Equations 1 and 2:

$$\frac{\partial u}{\partial x}=Sobel\left(u,dx=1,dy=0\right)$$ (1)

$$\frac{\partial v}{\partial y}=Sobel\left(v,dx=0,dy=1\right)$$ (2)

The local strain was defined by Equation 3:

$$\varepsilon =\frac{1}{2}\left(\frac{\partial u}{\partial x}+\frac{\partial v}{\partial y}\right)$$ (3)

A binary mask was generated from the polygon vertices defining the ROI on the grayscale image. Pixel values outside the ROI in the strain field were set to zero, retaining only the strain data within the ROI.

The overall strain magnitude for each frame was extracted by applying a least-squares linear fit to the displacement values within the ROI. Assuming a linear relationship between the displacement field and spatial coordinates, the system was modeled using Equations 4 and 5:

u=ax+by+c (4)

v=dx+ey+f (5)

The parameter vectors {a, b, c} and {d, e, f} were solved via the least-squares method, where parameters a and e represent the horizontal and vertical displacement gradients, respectively. The overall strain of the ROI was then expressed by Equation 6:

$$\varepsilon_{ROI}=\frac{1}{2}\left(a+e\right)$$ (6)

**FIGURE 2:**
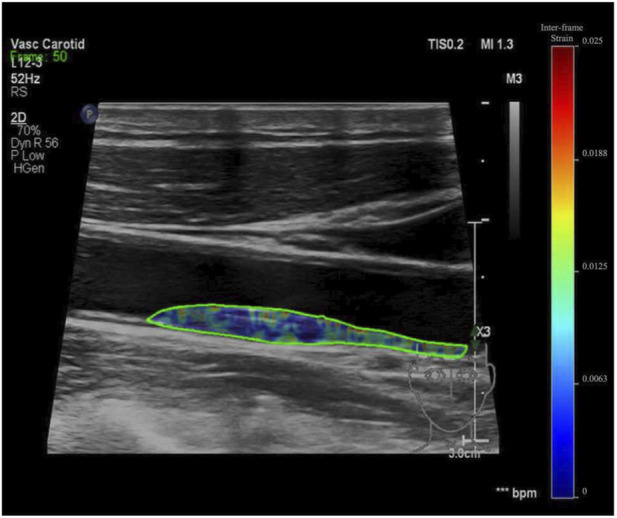
Region of interest and its inter-frame strain field contour map.

**FIGURE 3:**
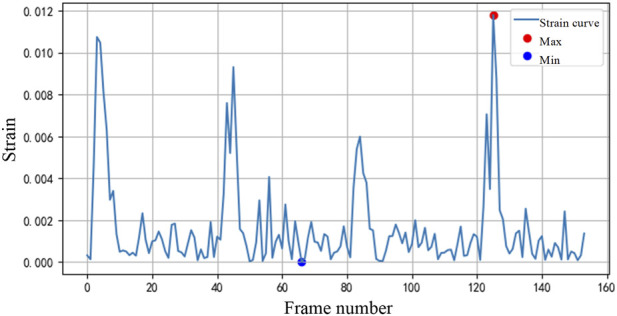
Diagram of strain quantification curves for adjacent frames of plaque 1-2 component regions.

**FIGURE 4:**
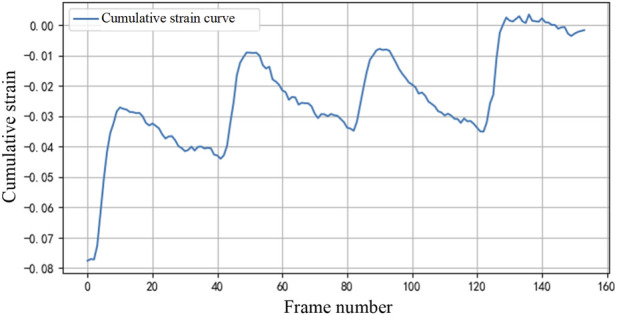
Accumulated curve of full-time-domain strain of plaque 1-2 component regions.

The strain field was normalized and mapped to a pseudo color table to generate a heatmap. This heatmap was overlaid onto the registered B-mode image, with colormap filling confined to the ROI to highlight the strain distribution within the plaque. This visualization uses a red-to-blue color gradient to distinguish high- and low-strain regions, enhancing visual contrast as shown in Figure 2. A frame-to-frame strain variation curve was plotted to reflect the dynamic changes in plaque component regional strain. Since the computed strain values can be positive or negative (representing expansion or contraction of the ROI, respectively). The negative values may lead to redundant results when comparing inter-frame strain results. Therefore, the absolute values of inter-frame strain were used to better observe waveform trends in the variation curve, as illustrated in Figure 3. Additionally, a cumulative strain curve was generated by summing the strain values from each starting frame to the end of the sequence, representing the accumulated strain per frame, which must include both positive and negative movements during the accumulation process. Thus, absolute value calculations are not applicable, and both positive and negative values must be retained. Since the cumulative strain for each frame is calculated over a cardiac cycle starting from that frame, the resulting value is negative, shown in Figure 4. The maximum inter-frame strain (MaxIS) value is defined as the maximum strain value of the plaque component region between any two frames within a cardiac cycle, which is used to quantify the maximum instantaneous elastic deformation capacity of the plaque component region. The mean inter-frame strain (MeanIS) value is defined as the average strain value of the plaque component region between any two frames within a cardiac cycle, which represents the average level of the instantaneous elastic deformation capacity of the plaque component region. The maximum cumulative strain (MaxCS) value is defined as the cumulative value with the largest absolute value of the inter-frame strain values of the plaque component region within a complete cardiac cycle, which is used to quantify the maximum elastic deformation capacity of the plaque component area throughout the entire cardiac cycle.

### 2.5 Data analysis

All statistical analyses were performed using MATLAB R2021a (MathWorks, Natick, MA, United States). In the correlation analysis, all plaque samples were regarded as independent. Pearson correlation analysis was applied to assess linear relationships between BP parameters and plaque component regional strain parameters. A P-value of less than 0.05 was considered statistically significant.

## 3 Results

To minimize subjective errors associated with manual annotation, the region of interest (ROI) delineation in this study was guided by the “Chinese expert consensus on ultrasound evaluation of vulnerable carotid plaque (2023 edition)” from the Chinese Medical Doctor Association Ultrasound Branch (Chinese Medical Doctor Association Ultrasound Branch, 2023). Plaques with relatively distinct echogenic features on carotid elastography images were selected for strain quantification to ensure the stability and repeatability of the results.

A total of 11 patients (seven males and four females) were included in this study, with ages ranging from 58 to 81 years. The systolic blood pressure (SBP) ranged from 115.0 to 135.5 mmHg, and the diastolic blood pressure (DBP) ranged from 60.00 to 85.50 mmHg. Detailed patient information and BP data are summarized in Table 1.

**TABLE 1:** Patient information.

Patients	Sex	Age (years)	SBP (mmHg)	DBP (mmHg)
1	Male	69	135.0	80.00
2	Male	58	115.0	62.50
3	Female	62	131.0	65.00
4	Male	69	135.5	75.50
5	Male	60	121.0	73.00
6	Female	70	133.0	83.90
7	Female	81	128.0	60.00
8	Male	69	124.0	72.00
9	Male	59	121.0	82.00
10	Male	69	131.0	85.50
11	Female	74	123.0	65.00

SBP, systolic blood pressure; DBP, diastolic blood pressure.

### 3.1 Significant variability in dynamic plaque component regional strain

Detailed results of the dynamic strain quantification for plaque component regions in the 11 patients are presented in Table 2. The MeanIS values across patients ranged from 0.0007 to 0.0045, representing a 542.8% variation among different plaques. More notably, the MaxIS values exhibited an even greater disparity, ranging from 0.0025 to 0.0454, showed a variation of up to 1716% across plaques. The strain parameters quantified from the 26 plaque component samples revealed marked inter-sample heterogeneity, strongly suggests distinctions in the mechanical response capacity of different plaque component regions when subjected to vascular wall stress.

**TABLE 2:** Inter-frame strain quantification results of carotid plaque component regions from 11 patients.

Patients	Plaques	SBP (mmHg)	DBP (mmHg)	PP (mmHg)	MeanIS	MaxIS
1	1–1	135	80	55	0.0027	0.0153
	1–2	135	80	55	0.0016	0.0118
	1–3	135	80	55	0.0026	0.0145
2	2–1	115	62.5	52.5	0.0019	0.0073
	2–2	115	62.5	52.5	0.0009	0.0081
3	3–1	131	65	66	0.0011	0.0052
	3–2	131	65	66	0.0015	0.0056
4	4–1	135.5	75.5	60	0.0023	0.0116
	4–2	135.5	75.5	60	0.0009	0.0047
5	5–1	121	73	48	0.0015	0.0075
	5–2	121	73	48	0.0011	0.0045
6	6–1	133	83.9	49.1	0.0010	0.0047
7	7–1	128	60	68	**0.0007**	0.0034
	7–2	128	60	68	**0.0007**	**0.0025**
	7–3	128	60	68	0.0015	0.0061
8	8–1	124	72	52	0.0011	0.0071
	8–2	124	72	52	0.0011	0.0054
9	9–1	121	82	39	0.0019	0.0071
	9–2	121	82	39	**0.0045**	**0.0454**
	9–3	121	82	39	0.0026	0.0153
10	10–1	131	85.5	45.5	0.0028	0.0114
	10–2	131	85.5	45.5	0.0015	0.0067
	10–3	131	85.5	45.5	0.0033	0.0128
11	11–1	123	65	58	0.0015	0.0069
	11–2	123	65	58	0.0013	0.0062
	11–3	123	65	58	0.0030	0.0153

SBP, systolic blood pressure; DBP, diastolic blood pressure; PP, pulse pressure; MeanIS, mean inter-frame strain; MaxIS, maximum inter-frame strain. Bold indicates the maximum and minimum values.

### 3.2 Cumulative strain analysis over the full time domain

When extending the analysis to cumulative strain over the entire acquisition time, the differences between plaque component regions became even more pronounced. The cumulative strain results for plaque component regions from the 11 patients are shown in Table 3. The quantified results indicated that the MaxCS values ranged from 0.0078 to 0.1948, reflecting a remarkable 2397% variation among different plaques. Plaques with high cumulative strain warrant particular attention. The plaque 9-2 from P9 ranked highest, with a MaxCS value of 0.1948, followed by the plaque 10-1 from P10 (MaxCS value: 0.1661). These plaques underwent substantial deformation accumulation under sustained vascular load, potentially indicating mechanical weakness in their internal structure. In contrast, the plaque 7-1 from P7 (MaxCS value: 0.0078) demonstrated comparatively better mechanical stability.

**TABLE 3:** The cumulative results of full-time domain strain of carotid plaque component regions in 11 patients.

Patients	Plaques	SBP (mmHg)	DBP (mmHg)	PP (mmHg)	MaxCS
1	1–1	135	80	55	0.1005
	1–2	135	80	55	0.0775
	1–3	135	80	55	0.0856
2	2–1	115	62.5	52.5	0.1017
	2–2	115	62.5	52.5	0.0166
3	3–1	131	65	66	0.0835
	3–2	131	65	66	0.0477
4	4–1	135.5	75.5	60	0.0489
	4–2	135.5	75.5	60	0.0227
5	5–1	121	73	48	0.0351
	5–2	121	73	48	0.0426
6	6–1	133	83.9	49.1	0.0203
7	7–1	128	60	68	**0.0078**
	7–2	128	60	68	0.0118
	7–3	128	60	68	0.0558
8	8–1	124	72	52	0.0199
	8–2	124	72	52	0.0352
9	9–1	121	82	39	0.0514
	9–2	121	82	39	**0.1948**
	9–3	121	82	39	0.1229
10	10–1	131	85.5	45.5	0.1661
	10–2	131	85.5	45.5	0.0537
	10–3	131	85.5	45.5	0.0689
11	11–1	123	65	58	0.0539
	11–2	123	65	58	0.0329
	11–3	123	65	58	0.0530

SBP, systolic blood pressure; DBP, diastolic blood pressure; PP, pulse pressure; MaxCS, maximum cumulative strain. Bold indicates the maximum and minimum values.

### 3.3 Correlation analysis between blood pressure and carotid plaque component regional strain

Pearson correlation coefficients (r) were calculated to assess the linear relationships between SBP and DBP and plaque component regional strain parameters (mean strain and maximum strain). The results are summarized in Table 4. Based on the correlation coefficients in Table 4, it can be observed that SBP exhibited a weak positive correlation with the MeanIS of plaque composition (r = 0.0183), and weak negative correlations with both the MaxIS and the MaxCS of plaque component regions (r = −0.0900, −0.0148, respectively). In contrast, DBP showed moderate positive correlations with the MeanIS and the MaxCS of plaque composition (r = 0.5121, 0.4606), and a weak positive correlation with the MaxIS (r = 0.3977), all of which reached statistical significance. Meanwhile, the pulse pressure (PP) demonstrated moderate negative correlations with all three strain indices (r = −0.5043, −0.4650, −0.4757), showing statistical significance even with a small sample size.

**TABLE 4:** Correlation results between blood pressure and plaque strain in 11 patients.

Variables	Pearson correlation coefficient (*r*)	P-value (*p*)
SBP vs. MeanIS	0.0183	0.9292
SBP vs. MaxIS	−0.0900	0.6618
SBP vs. MaxCS	−0.0148	0.9427
DBP vs. MeanIS	0.5121	**0.0075**
DBP vs. MaxIS	0.3977	**0.0442**
DBP vs. MaxCS	0.4606	**0.0179**
PP vs. MeanIS	−0.5043	**0.0086**
PP vs. MaxIS	−0.4650	**0.0167**
PP vs. MaxCS	−0.4757	**0.0140**

SBP, systolic blood pressure; DBP, diastolic blood pressure; PP, pulse pressure; MeanIS, mean inter-frame strain; MaxIS, maximum inter-frame strain; MaxCS, maximum cumulative strain. Bold indicates that the p-value is less than 0.05.

## 4 Discussion

### 4.1 Pathophysiological implications of inter-patient variability in dynamic plaque component regional strain

Our results demonstrate substantial mechanical heterogeneity in carotid plaque component regions among patients. The observed variations in strain parameters exceeding 500%, with cumulative strain differences of up to 2400%, reflect the underlying complexity and diversity of plaque composition (Naghavi et al., 2003; Hansen et al., 2016). Biomechanical and histopathological studies have confirmed that high-strain regions often correlate strongly with lipid-rich necrotic cores, thin fibrous caps, and active inflammatory activity—all hallmarks of plaque vulnerability (Irons et al., 2023). Conversely, low strain typically indicates dense fibrous collagen or calcification, signifying plaque stability. Therefore, the quantitative, visual representation of plaque component regional strain provided by this study holds promise as a mechanical biopsy map, potentially serving as an objective imaging basis for clinicians to differentiate plaque vulnerability and optimize treatment strategies. The high accumulation of strain at the lipid core has the potential to predict the risk of no reflux phenomenon during holding. The high accumulation of strain in the lipid-rich areas may serve as a candidate imaging marker for predicting perioperative complications.

### 4.2 Association between blood pressure and plaque component regional strain: exploratory findings

This study elucidates the complex relationships between DBP components and the biomechanical behavior of carotid plaque through quantitative strain analysis. Our findings reveal a weak and inconsistent correlation between SBP and various plaque strain metrics. Specifically, SBP showed a negligible positive correlation with MeanIS (r = 0.0183) but weak negative correlations with both MaxIS and MaxCS (r = −0.0900 and −0.0148, respectively). This pattern challenges the conventional assumption that elevated SBP directly increases plaque deformation. In this dataset, SBP varies only modestly (127.05 ± 6.68 mmHg), while DBP varies more meaningfully (73.13 ± 9.05 mmHg). Therefore, the lack of correlation between SBP and strain may be partly attributable to the relatively narrow range of systolic values within this cohort. Beyond potential sample size limitations, this discrepancy may stem from altered pressure-strain relationships in chronic hypertension, where compensatory vascular remodeling and plaque calcification might suppress strain capacity beyond a certain pressure threshold (Okuno et al., 2023; Christensen et al., 2024). This interpretation aligns with clinical observations of paradoxically reduced plaque activity in patients with severe hypertension (Pernomian et al., 2025; Thenappan et al., 2018). Nevertheless, as the primary cyclic load on the arterial wall, SBP likely still contributes to cumulative intimal damage within plaques via peak stress, potentially leading to fibrous cap fatigue and morphological changes over time.

In contrast, DBP demonstrated significant positive correlations with all strain indicators, suggesting its possible role in maintaining the continuous deformation of the plaque. Biomechanically, DBP determines the static load on the vessel wall. Elevated DBP may impose continuous tensile stress on plaque component regions, promoting micro-crack initiation and propagation, thereby maintaining the plaque under high tension. This sustained high-stress environment constitutes a critical biomechanical basis for plaque rupture. Furthermore, lower DBP might indirectly modulate plaque mechanical properties by reducing shear stress and affecting endothelial function and extracellular matrix composition. In specific populations, such as elderly patients with isolated systolic hypertension, DBP could serve as a valuable biomarker for assessing plaque vulnerability.

Notably, pulse pressure (PP) showed moderate negative correlations with all strain metrics, suggesting that reduced pulsatile load coincides with increased plaque strain. This apparent paradox may reflect a biomechanical compensatory mechanism in the context of vascular stiffening: although BP variability diminishes as arterial compliance decreases, the local mechanical stress on the plaque may actually increase due to elevated vessel wall stiffness. Additionally, increased PP can amplify wave reflections, creating localized stress concentrations at vulnerable sites like the plaque shoulder, thereby exacerbating strain gradients and structural instability (Zhao et al., 2024).

In conclusion, these exploratory results identify DBP and PP as potentially relevant mechanical factors associated with plaque deformation. These findings provide quantitative biomechanical observations that may support further investigation of blood pressure dynamics in plaque vulnerability. The MaxIS is a quantitative indicator of the elastic deformation capacity of the plaque component region between every two frames, and the MaxCS is a quantitative indicator of the elastic deformation capacity of the plaque component region in one cardiac cycle. In the future, the MaxIS and MaxCS combined with blood pressure data can form a strain threshold of potential high risk, which is used to determine whether to enhance statin treatment or more aggressive antihypertensive treatment in clinical practice. Future studies integrating compositional imaging and computational modeling are warranted to further elucidate the intricate interactions between plaque structure and its mechanical environment, which may offer new perspectives for individualized cardiovascular risk prevention and management.

### 4.3 Carotid plaque component regional strain quantification: methodology and clinical relevance

This study established an automated pipeline for quantifying dynamic strain in plaque component regions by integrating dynamic vascular imaging with advanced image processing, achieving dynamic, quantitative, and pixel-level precision. The application of the Farnebäck dense optical flow method provided substantially higher information density than sparse optical flow techniques, enabling detailed characterization of micro-strain distributions within plaque components. An innovative dynamic ROI update mechanism effectively mitigated drift issues inherent in long-sequence tracking. Finally, estimating the overall ROI strain via least-squares fitting robustly suppressed noise interference, yielding stable and reliable mechanical characterization metrics. This framework presents a feasible technical pathway for translating research-grade algorithms into a potential clinical tool.

### 4.4 Limitations

This study has several limitations. First, its exploratory nature and small sample size may limit the statistical power and generalizability of the findings. In the correlation analysis, all plaque samples were regarded as independent observations, which could not explain patient clustering and limited our interpretation of the partial statistical significance of the results. Second, although the initial ROI delineation was performed by an experienced physician who was also blinded to the patients’ clinical and blood pressure data, it remained a manual process subject to inherent subjectivity. The subjective influence on image quality and ROI repeatability is an important limitation. Third, *in vivo* imaging data lacked direct histopathological correlation, preventing verification of the specific tissue composition corresponding to high-strain regions. Based on the inherent advantage of ultrasound in classifying plaque components, and with the acquisition of more patient data, our future research will explicitly discuss the conditions of different plaque components in the plaque component regions. This will be a new and meaningful result in the future. Fourth, the accuracy of the optical flow method is constrained by image quality and frame rate, necessitating preliminary screening of patient data at the current stage. Finally, we selected patients with type 2 diabetes because the phenotype of vulnerable plaques is more common among this group of patients. This limitation restricts the applicability of the research results to non-diabetic populations. Future studies should include a broader sample group.

## 5 Conclusion

This study initially demonstrated the mechanical heterogeneity among the plaque component regions. The exploratory analysis indicated that diastolic blood pressure and pulse pressure may be potential mechanical factors associated with plaque deformation. This study initially demonstrated the potential of using dynamic ultrasound imaging to quantitatively measure the dynamic strain of plaque component regions. These findings may provide important mechanical insights for individualized cardiovascular risk assessment and precise intervention, though further validation in larger cohorts is needed.

## Data Availability

The raw data supporting the conclusions of this article will be made available by the authors, without undue reservation.
